# Simulation and Optimization Studies of the LHCb Beetle Readout ASIC and Machine Learning Approach for Pulse Shape Reconstruction

**DOI:** 10.3390/s21186075

**Published:** 2021-09-10

**Authors:** Pawel Kopciewicz, Kazuyoshi Carvalho Akiba, Tomasz Szumlak, Sebastian Sitko, William Barter, Jan Buytaert, Lars Eklund, Karol Hennessy, Patrick Koppenburg, Thomas Latham, Maciej Majewski, Agnieszka Oblakowska-Mucha, Chris Parkes, Wenbin Qian, Jaap Velthuis, Mark Williams

**Affiliations:** 1 Department of Particle Interactions and Detection Techniques, Faculty of Physics and Applied Computer Science, AGH University of Science and Technology, 30-059 Krakow, Poland; Szumlak@agh.edu.pl (T.S.); Sebastian.Sitko@fis.agh.edu.pl (S.S.); Maciej.Witold.Majewski@cern.ch (M.M.); AMucha@agh.edu.pl (A.O.-M.); 2 Nikhef National Institute for Subatomic Physics, 1098 XG Amsterdam, The Netherlands; Kazu.Akiba@cern.ch (K.C.A.); Patrick.Koppenburg@cern.ch (P.K.); 3 Department of Physics, Imperial College, London SW7 2AZ, UK; William.Barter@cern.ch; 4 European Organization for Nuclear Research (CERN), 1211 Geneva, Switzerland; Jan.Buytaert@cern.ch; 5 School of Physics and Astronomy, University of Glasgow, Glasgow G12 8QQ, UK; Lars.Eklund@cern.ch; 6 Department of Physics and Astronomy, Uppsala University, 751 05 Uppsala, Sweden; 7 Oliver Lodge Laboratory, University of Liverpool, Liverpool L69 7ZE, UK; Karol.Hennessy@cern.ch; 8 Department of Physics, University of Warwick, Warwick CV4 7AL, UK; T.Latham@warwick.ac.uk; 9 School of Physics and Astronomy, University of Manchester, Manchester M13 9PL, UK; Chris.Parkes@cern.ch; 10 University of Chinese Academy of Sciences, Beijing 100049, China; Wenbin.Qian@cern.ch; 11 H.H. Wills Physics Laboratory, University of Bristol, Bristol BS8 1TH, UK; Jaap.Velthuis@bris.ac.uk; 12 School of Physics and Astronomy, University of Edinburgh, Edinburgh EH8 9YL, UK; Mark.Williams@cern.ch

**Keywords:** particle tracking detectors, sensor optimization, readout systems

## Abstract

The optimization of the Beetle readout ASIC and the performance of the software for the signal processing based on machine learning methods are presented. The Beetle readout chip was developed for the LHCb (Large Hadron Collider beauty) tracking detectors and was used in the VELO (Vertex Locator) during Run 1 and 2 of LHC data taking. The VELO, surrounding the LHC beam crossing region, was a leading part of the LHCb tracking system. The Beetle chip was used to read out the signal from silicon microstrips, integrating and amplifying it. The studies presented in this paper cover the optimization of its electronic configuration to achieve the lower power consumption footprint and the lower operational temperature of the sensors, while maintaining a good condition of the analogue response of the whole chip. The studies have shown that optimizing the operational temperature is possible and can be beneficial when the detector is highly irradiated. Even a single degree drop in silicon temperature can result in a significant reduction in the leakage current. Similar studies are being performed for the future silicon tracker, the Upstream Tracker (UT), which will start operating at LHC in 2021. It is expected that the inner part of the UT detector will suffer radiation damage similar to the most irradiated VELO sensors in Run 2. In the course of analysis we also developed a general approach for the pulse shape reconstruction using an ANN approach. This technique can be reused in case of any type of front-end readout chip.

## 1. Introduction

The Beetle ASIC [1] was a front-end electronic readout chip used at the LHC (Large Hadron Collider) at CERN in the LHCb (Large Hadron Collider beauty) experiment [2] in the VELO (Vertex Locator) [3,4] since the beginning of the experiment in 2010. The ASIC had to cope with high radiation doses [5] as the readout stations were located in close proximity to the LHC beam crossing region. Over its life span, the VELO silicon sensors were irradiated enough that at the end of the last LHC run campaign, in December 2018, the irradiation effects in VELO [6], such as the thermal runaway [7] (driven by the increase in leakage current) were considered a major challenge for the detector maintenance and physics performance [8]. To mitigate these adversarial effects, a number of sensors had to be operated at lower bias voltages. Addressing this issue, the studies of re-optimization of the ASIC configuration using computational intelligence techniques, focusing on reducing the power consumption and its operational temperature were proposed. The artificial intelligence methods and machine learning applications in general became more popular in the high energy physics field recently [9,10], in physics at the LHC specific environment [11], and the silicon detectors [12]. The intelligent methods were also employed for supporting the analogue signal processing and discrimination also in germanium semiconductor detectors [13]. In this paper, the optimization process and the actual data taking were backed by modeling of the analogue pulse shape using a machine learning approach. The optimization focused mainly on the ASIC’s front-end configuration settings. It was initially expected that the reduction in power consumption of the ASIC is likely to decrease its readout performance and influence the analogue response of the whole chip. When the initial studies were conducted, it was discovered that some of the proposed configurations brought not only the lower temperature but also the better condition of the analogue pulse shape in front-end electronics. It proved the default configuration for irradiated ASIC to be no longer optimal in terms of the readout performance. After the studies, a suite of new ASIC settings was proposed to improve the analogue pulse shape conditions and reduce the temperature.

The paper is organized as follows. The complete studies of optimization of the Beetle readout ASIC are presented in Section 3, including the machine learning contribution to the pulse shape reconstruction. They are preceded by the detailed description of the LHCb experiment, the VELO detector and Beetle ASIC in Section 2. Finally, conclusions are given in Section 4, as are the further applications of this research. The silicon detectors at LHC will have to cope with increasing luminosity in the next decades. Given this, the studies can be beneficial for the further development of semiconductor tracking detectors. In particular, the Upstream Tracker application is proposed, a silicon detector to be part of the LHCb Upgrade I program [14], which will operate at LHC from 2021 to approximately 2030, as its readout system is similar to the VELO one [15,16].

## 2. Materials and Methods

The LHCb (Large Hadron Collider beauty) detector [17] is a single-arm forward spectrometer installed at the LHC to study heavy flavor physics. The research program of LHCb focuses mainly on searching for evidence of New Physics in the charge-parity violation (CPV) phenomena and in rare heavy meson decays. Such studies require exploring large data samples with different decay modes containing b- and c-quarks. The LHCb acceptance in pseudorapidity (pseudorapidity, η, is defined as η=−ln[ tan(θ/2) ], where θ is the polar angle of the track with respect to the beam) covers the region of 1.8 < η < 4.9. The spectrometer consists of a vertex detector, tracking stations, magnet, RICH detector, calorimeters, and muon detectors [18]. The first and second phases of LHCb operation (known as the LHC Run 1 and 2) took place from 2010 to 2012 and from 2015 to 2018, respectively. Since the end of Run 2, the LHCb detector is being upgraded to enable the detector to collect data with an increased number of concurrent collisions (pile-up) and, consequently, to augment the physical data collected by the experiment each year. The LHC Run 3 is scheduled to start in 2022.

**Figure 1:**
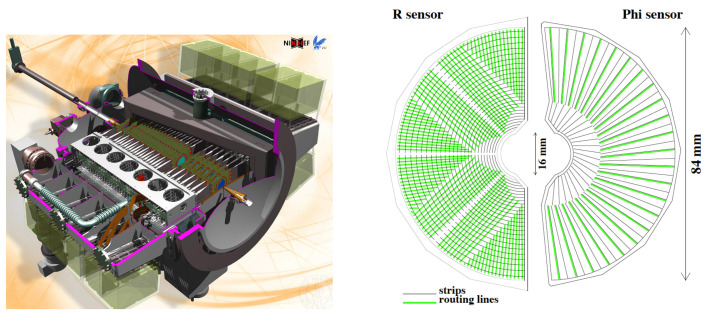
A visualization of the Vertex Locator showing the detector stations placed along the beam line (on the **left**). The RF foil and cooling mechanism are also visible. The scheme of R and Φ type of sensor (on the **right**) [19].

The Vertex Locator (VELO) is a silicon tracking detector surrounding the beam intersection region in the LHCb spectrometer [4]. The main goal of the VELO is to reconstruct the primary proton-proton collision vertices and the secondary decay vertices. It also takes part in lifetime measurements and significantly contributes to the LHCb event trigger. The angular coverage of the VELO allows the reconstruction of tracks from particles moving both forward (downstream) and backward (upstream). During LHC Run 1 and 2, the VELO was a silicon microstrip detector featuring two types of sensors, R and Φ, with silicon strips arranged radially and transversely, respectively, and a minimum pitch of 37 μm. The VELO consists of two halves and 21 stations placed alongside the beam, with modules composed of one R and one Φ type sensor. In addition, two pile-up stations employing only R sensors are used for upstream tracking. During the injection of the LHC beams, the two halves of the VELO are moved apart while the beams stabilize, to protect the detector from potential damage. Once the beams are stabilized, the halves move to the closed position. The stations are installed in a secondary vacuum, separated from the primary vacuum by a thin RF foil [6] that shields the sensor assemblies from electromagnetic radiation. A schematic of the VELO detector, as well as the R and Φ sensors, are presented in Figure 1.

An effective readout frequency of 1.1 MHz is achievable at the output of the L0 hardware trigger [20]. The L0 trigger is followed by the software HLT (High-Level Trigger), which outputs data with a frequency of 100 kHz and yields events that are further directed to the CPU farm for subsequent processing. The pile-up sensors readout at 40 MHz and contribute to the L0 trigger.

The VELO detector is one of the key LHCb spectrometer components that is being replaced for the LHCb Upgrade I [21]. The silicon strip sensors are going to be replaced by silicon pixel ones [22] with new trigger-less readout electronics that can cope with a 40 MHz readout rate (which is the LHC machine clock). There are two key factors driving the upgrade project. Firstly, we want to exploit better the machine potential and collect larger data samples while running at five times higher instantaneous luminosity than during Run 2. The hardware-based trigger layer (during Run 1 and Run 2) constituted a barrier that limited the amount of data taken [23]. Secondly, most of the detector’s sub-systems would not operate at the higher luminosity due to obsolete readout electronics and the radiation-induced damage they sustained during Run 1 and Run 2 data taking [24,25,26]. These goals cannot be achieved only by redesigning the respective sub-detectors of the LHCb spectrometer alone [27]. The critical part of the upgrade is an innovative, flexible fully-software trigger system that can maximize efficiencies for hadronic decay channels, requiring the new trigger-less front-end electronics capable of reading out the full detector at 40 MHz [28,29]. The upgraded VELO will be adjusted to that readout [30,31]. One should note that the principal role of the VELO will remain the same. The installation of the new detector is progressing steadily and will be finished by early 2022.

### 2.1. The Beetle Readout Chip

**Figure 2:**
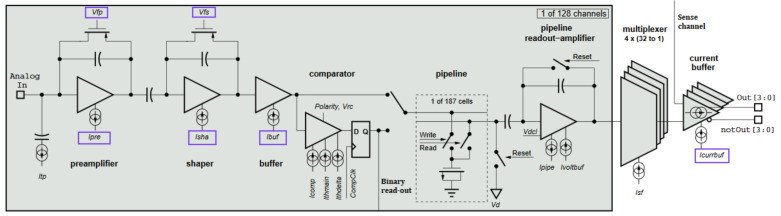
Single channel circuit scheme of the Beetle chip. The bias voltages and currents related to the analogue pulse shape are indicated by violet rectangles [1].

The VELO readout is operated by a dedicated ASIC, the Beetle chip, an electronic readout component equipped with both analogue and digital modes of signal processing [1]. The chip combines 128 independent input channels. Each front-end channel consists of a charge-sensitive preamplifier, a CR-RC pulse shaper, and a buffer. The circuit converts the integrated input charge into a semi-gaussian pulse. The pulse shape can be changed by modifying the electronic parameters of the front-end circuit, e.g., the preamplifier discharge current. Digital-to-Analogue Converters (DAC) embedded in the chip generate bias currents and voltages with 8-bit resolution. Customizable front-end settings allow the chip to be used in various applications with different signal processing requirements. These include balancing the expected pulse rise-time and limiting the signal remainder in consecutive clock cycles corresponding to the consecutive beam crossings. This balancing procedure was studied in detail at the beginning of the LHCb experiment operation since the test-beam studies showed that a not optimized pulse-shape may lead to spurious, fake hits in the detector. The electronic scheme of the Beetle chip is shown in Figure 2.

The analogue buffer output is sampled by a 40 MHz clock into an analogue pipeline memory that stores the events with a maximal latency of 160 clock intervals. Upon receipt of a trigger, the event is marked for readout; up to 16 triggers can be queued. The readout of a single trigger takes 900 ns, in which the values stored in the pipeline memory are serialized by a multiplexer, and sent from the chip. An alternative path directs a signal from the analogue buffer into the signal discriminator that serves the binary readout.

Each Beetle channel is equipped with an input charge generator. Studies presented in this paper explore the pulse shape behavior as a function of the front-end configuration, searching for a new set of optimal parameters to configure the readout electronics after years of working in the intense hadronic radiation environment at the LHCb detector. The procedure applied to reconstruct the pulse shape is described in Section 2.2, and the various different settings that were used to measure and reconstruct the pulse shapes are discussed in Section 3.

### 2.2. Pulse Shape Reconstruction

To optimize the synchronicity of the Beetle digitization and to maximize the signal to noise ratio on the readout two delay parameters are relevant. The time of the sampling of the pulse inside the front-end electronics and the time of the digitization of the output pulse relative to the clock of the front-end electronics. During the data taking period, these two parameters were quickly adjusted to give the highest possible signal to noise ratio. The digitization time was scanned by changing the input clock of the Beetle chips in steps of 1 ns. The result of such a scan can be rearranged to the output signal of the Beetles as a function of the readout time, as the 25 ns readout is multiplexed into 32 channels. A test pulse signal injected on a few channels helps to adjust the sampling point.

Studying the pulse shape of the front-end response requires changing either the time each particle crosses the detector or the time of the entire electronic readout with respect to the time of the incident particle. Since changing the beam crossing time is not practical and would be hard to achieve, the proposed solution was to delay the clock of the readout supervisor board, which provides the Beetle clock. Applying consecutive delays to the clock makes the reconstruction of a signal shape attainable. However, it is not possible to measure delays larger than the clock width of 25 ns as they will wrap around to the next clock cycle. The analogue response pulse–shape time evolution of the Beetle is longer than one clock cycle. Therefore, it was decided to send seven consecutive readout triggers, which read out seven consecutive pipeline memory positions containing the integrated charge of the sampled signal at 25 ns intervals. In this way, the charge measured at several consecutive clock cycles is obtained and, by changing the delay of each clock by small steps, the pulse shape can be reconstructed. In order to recreate the pulse shape using MIPs (Minimum Ionizing Particles) [32], a signal maximum on the expected pipeline position for the peak is searched on all of the strips of the given sensor. A pedestal is calculated individually for each channel by averaging many samples.

Next, the ADC (Analog to Digital Counts) value is checked to ascertain whether it passes a certain threshold, calculated based on the noise value after the pedestal subtraction. A sufficient estimate of the noise can be made by taking the width of the measured ADC distribution in the absence of the signal. This condition is satisfied for the first samples when the charge collected due to incident particles can be neglected. Hence, a noise is estimated as an average among the width of the first four samples. When the actual signal shape is scaled by the inverse of this noise estimate, one obtains the signal to noise pulse shape in signal to noise ratio units.

**Figure 3:**
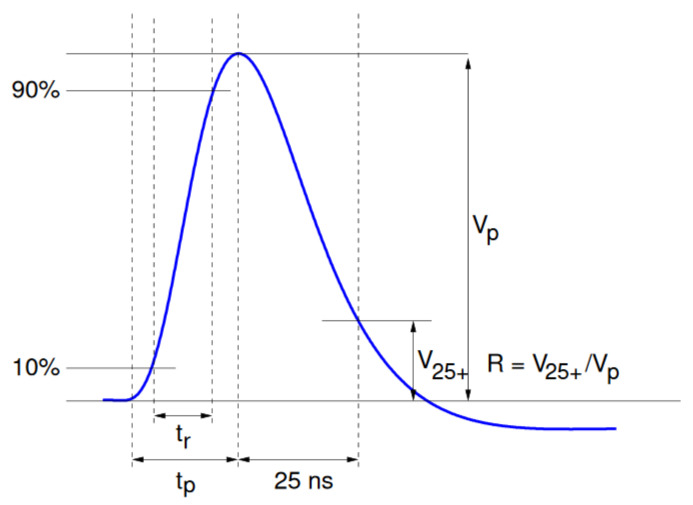
The signal pulse shape seen at the output of the front-end. The amplitude of the signal is directly proportional to the charge in the channel [1].

The pulse has an asymmetric semi-gaussian shape that can be described by the following set of parameters: the height of the pulse $V_{p}$; the rise time $t_{r}$ measured on the rising edge between 10% and 90% of the total height; the total peaking time $t_{p}$ measured for the whole rising edge; and the signal remainder or a spill-over *R* defined as the ratio between the signal height 25 ns after the peak $V_{25+}$ and the total height of the pulse. Those parameters are visualized in Figure 3. According to this definition, for the correctly calibrated detector, the upper limit for the spill-over ratio is one. One could also, by analogy, define a pre-spill with the similar criterion given. Pre-spill is a ratio between the signal height 25 ns before the peak $V_{25-}$ and the total height of the pulse. Following the same logic, an undershoot can be defined as the residual charge 50 ns after the desired sampling point, which will eventually tend towards the initial pedestal value. The nominal amplifier discharge current cannot discharge the whole signal in a single 25 ns clock period. The signal remainders are sampled as well and can contribute to the signal observed on the same strip in the next event. This so-called spill-over charge has a major impact on the VELO hit efficiency due to the generation of fake hits and is one of the main reasons for the modeling studies presented in this paper. A large fake hits stream would introduce disturbances in the experiment’s data acquisition system, produce busier events and make the track reconstruction procedure less effective.

An emulation of the complete signal processing can be performed in Vetra [33], a dedicated software developed for the LHCb silicon detectors under the experiment-specific environment [34]. One of the main features of Vetra is the sensor readout simulation, including clustering algorithms and association of hits to tracks. Simulation software can be used in efficiency studies, especially when exploring data acquisition-related figures-of-merit that are not directly measurable in the real detector, such as the impact of spill-over hits on the track reconstruction efficiency.

### 2.3. Modeling the Pulse Shape

Modeling the front-end response is a vital component in the simulation of signal processing. The analogue pulse shape seen at the output of the front-end is a function of time and electronic set-up, and it cannot be expressed directly by an analytic formula. A pulse model, such as a modified gaussian or a polynomial construct, has to be used.

The magnitude of the pulse is proportional to the injected charge, and the pulse always keeps its shape due to the linearity of the front-end. The circuit is linear (<5% deviation) for a charge of up to 110 ke${}^{-}$, which is five times the charge of an MIP in 300 μm silicon. For convenience, the pulse shape is presented in units of signal to noise ratio, where the noise is calculated as a standard deviation of the given channel pedestal. Alternatively, the pulse amplitude can be calibrated in terms of the number of electrons generated in the sensor. The pulse that is reconstructed using the method described in the previous section is a discrete curve, since the smallest possible interval for shifting the clock was limited to 0.5 ns. In order to establish a continuous curve, the discrete curve has to be interpolated. The analytic approach, such as fitting polynomial compositions, is troublesome and not practical. It was found that in order to obtain a smooth interpolation in our case, four different six-degree polynomials have to be incorporated into the fit. An increase in the degree of the polynomials can reduce that number to three, which simplifies the model, but creates the unfavourable peculiarity of varying the ratio of consecutive polynomial coefficients over several orders of magnitude, resulting in diminished stability of the model. For the most part of Run 1 and 2, a cubic spline method was used. The cubic spline [35] is a commonly used technique for interpolating the shape of non-analytic functions, even though it has some disadvantages with respect to other methods. Cubic spline guarantees that all data points are perfectly included in the curve. No variance can be calculated in such an approach. Moreover, even single-point fluctuation from the real quantity strongly affects the shape of the curve. Spline interpolates data in the form of a big matrix of coefficients of cubic polynomials and borders of their domains what makes it tough to persist. For the most time, these drawbacks were considered minor, as the expected simulation uncertainty was low. Later studies showed, however, that such an approach yields more false events than those which occur in the detector in reality. Replacing the cubic spline method was followed by the choice of other approximation methods, and the higher-order polynomials and neural networks were taken into account. Ultimately, as the signal shape required a merge of three different polynomials, the examined model turned out to be hard to tackle, and a feed-forward neural network model was chosen instead.

An appropriately trained neural network can be used as an alternative approach for modeling the pulse shape that is free of the negative aspects of the models based on various polynomials considered above. Our studies show that a feed-forward neural network is a flexible and universal approach for the pulse shape approximation. The proper training of such a network requires constructing training, validation and test data sets. The data exploration phase was performed by analysis of the properties of the real pulse shapes. A dedicated procedure was prepared, where test pulses were injected to all strips of one R and one Φ type sensor in the real detector, and the test pulse reconstruction procedure described in Section 2 was applied. Next, the mean pulse shape over the 2048 channels of the R sensor and the 2048 channels of the Φ sensor was evaluated, and the two extreme cases were chosen as upper- and lower-boundary curves, respectively, so that no test pulses were rejected. The boundaries were further used to measure the pedestal spread for data augmentation. The measured width of the pulse shape curves spread was close to one ADC count, which corresponds to approximately 2.5% of the pulse maximal amplitude, and was approximately constant for all sampling points. Based on these results, a data augmentation procedure was prepared where a large number of pulse shape curves were drawn (represented as a vector of time stamps and corresponding values) by applying a random shift for all values of the mean pulse shape across all time stamp points. The shift was chosen randomly using a flat distribution defined by the measured pulse shape curves spread. Eventually, a sample of 25,000 such drawn pulses was prepared and divided into training, validation and test sets. The training set comprised 50% of all events, and the rest was split evenly into the validation and test ones.

In our analysis, the ROOT implementation of the neural network (TMultiLayerPerceptron [36]) was used, and the fit quality was evaluated using the absolute residuals between the model output and the average of all of the pulses used in the training process. The standard back-propagation learning algorithm was used for training. The optimal structure of the neural network, applied for the analogue pulse shape estimation, was studied using the validation set. Three different architectures were considered with one, two and many hidden layers and with a different number of neurons (nine distinct models were used). It was found that the best results, in our case, were obtained using a network with only one hidden layer and six neurons inside it. The model had the one-dimensional input of the time stamp, while the network processed it further and calculated the amplitude of the standardized shape.

**Figure 4:**
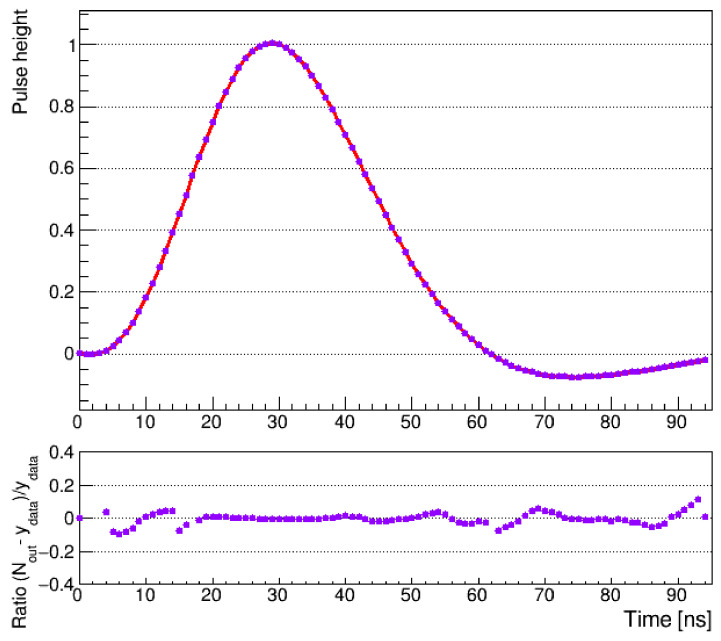
The pulse shape recreated by the neural network along with the ratio of the residuals $N_{out}-y_{data}$ to the mean pulse shape $y_{data}$ representing the uncertainty of the estimated pulse shape.

**Figure 5:**
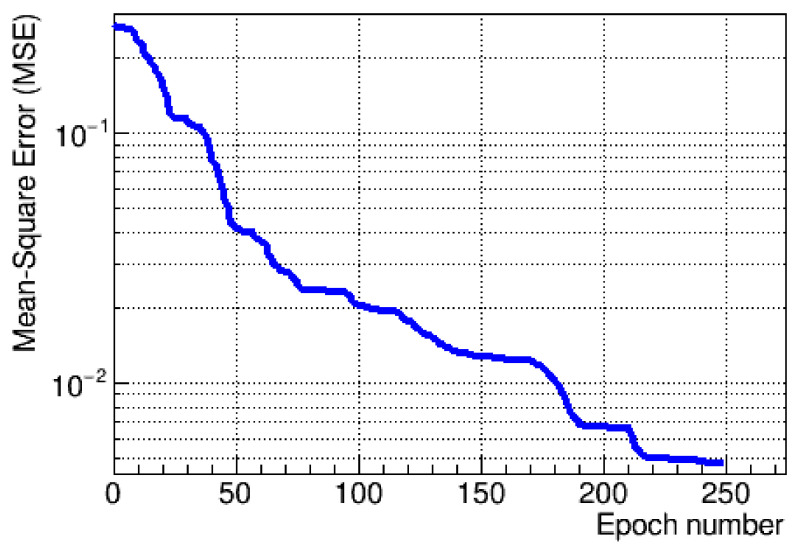
The gradient descent for the training process of the neural network.

The fit was found to be stable and repeatable, with a typical pattern of residuals distributed around the calculated fit for the testing data set. The fit, along with a ratio of the residuals to the mean pulse shape, is shown in Figure 4. The fit stability was studied as a function of the number of epochs for both training and test data sets, which showed a constant drop of the loss function without oscillations, with the gradual descent presented in Figure 5.

Such an approach results in a universal tool for modeling the pulse shape that was incorporated in both the full LHCb simulation software and the VELO monitoring platform Vetra and used for further studies on the optimization of the pulse shape presented in the following section. The simulation uses the mean measured shape from different Beetles to represent all readout chips and individually adds channel-specific properties, such as the strip capacitance.

## 3. Results

Since the temperature of the sensor is related to the power dissipation in the electronics, lowering the power consumption can provide enough of a safety margin to the point of thermal runaway. Therefore, as already stated in Section 1, it was decided to optimize the electronic settings in order to possibly mitigate the risk of thermal runaway.

During the studies, the following six specific electronic parameters were taken into account: the preamplifier and shaper bias currents, Ipre and Isha; the gate voltage of preamplifier and shaper feedback p-type MOS transistors, $V_{\mathrm{fp}}$ and $V_{\mathrm{fs}}$; the buffer bias current, $I_{\mathrm{curr}}$; and the memory readout amplifier bias current, icurrbuf. They are indicated in Figure 2 by violet rectangles. Their impact on the signal processing is complex but can be estimated according to the front-end specification [1]. A change of the nominal configuration can improve some of the readout properties, but usually at the cost of the power consumption or the other stages of the readout. Given this, the idea was to try to slightly decrease the values of the parameters to lower the power consumption of the electronics, balancing on the edge of maintaining the pulse shape. During the studies, a bunch of new proposed configuration settings were proposed. For reference, the default configuration of the Beetle’s nominal settings was used.

**Figure 6:**
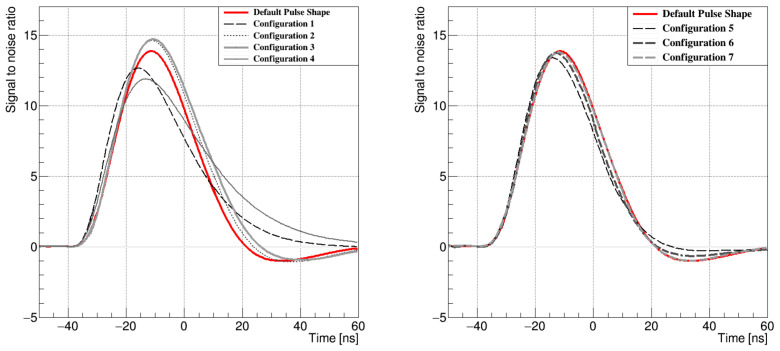
The first set of the pulse shapes with the configurations 1–4 (**left**) and the second set with configurations 5–7 (**right**).

The first attempt of the test pulse studies during a technical stop of the LHCb detector covered ten different configurations. Based on the analysis of the first set of configurations, a second set was proposed, and eight additional configurations were chosen to be measured at the earliest opportunity. All of the tested settings were expected to lower the power consumption of the electronics. The first attempt did not develop the settings that would fulfil the given criterion of reducing the power consumption with at least preservation of the pulse shape characteristics, particularly the remainder R, being accountable for spill-over events. Although the power consumption was slightly lowered, the measured pulse shapes were longer and wider than the default one. However, the second attempt was more successful and resulted in two settings that, apart from power consumption, improved the pulse conditions as well. The four best pulses from the first set of configurations and three from the second set were compared to the default shape and are presented in Figure 6. The values of the parameters in each configuration are given Table 1.

The study of the spill-over hit participation for all tested shapes was performed with the Vetra software platform using raw proton–proton collision data. In this context, raw data are those that pass only a minimal trigger selection criteria. The contribution from spill-over events is associated with the delay between the signal peak and the event sampling point. When no delay is applied and the signal is sampled at its peak, the spill-over in the next event can be very substantial. Applying a delay can lower the signal to noise ratio of the signal peak and decrease a further contribution of spill-over events, resulting in a higher signal to noise ratio in total. After measurements, where the overall conditions of the pulse shapes were taken into account, it was found that the best shape was achieved with Configuration 6. The optimal latency for that was found to be 4.5 ns. The preamplifier bias current that provides the preamplifier’s gain was reduced by around 10% of its initial nominal value, from 600 μA to 536 μA. Combined with a slight increase in the shaper bias current, the pulse turned out to be shorter, with only a minimal decrease in the peak amplitude. The improved pulse shape was subsequently implemented in the LHCb simulation software. Detailed studies with simulated data samples showed that the signal purity increased by 4.8% (from 0.669 to 0.702) compared to the default configuration, where the purity is defined as a ratio of the number of true hits to the number of true and spill-over hits combined.

The pulse shape scan procedure discussed in previous sections was performed with a constant input charge. In the normal detector operation, the charge seen at the input channel is not constant and is distributed according to a Landau convoluted with a Gaussian, provided the thickness of the sensor is small enough [37]. The charge-sensitive preamplifier at the front-end provides a linear gain in relation to the injected charge. Therefore, the amplitude of the reconstructed pulse forms the Gauss–Landau distribution as well.

**Figure 7:**
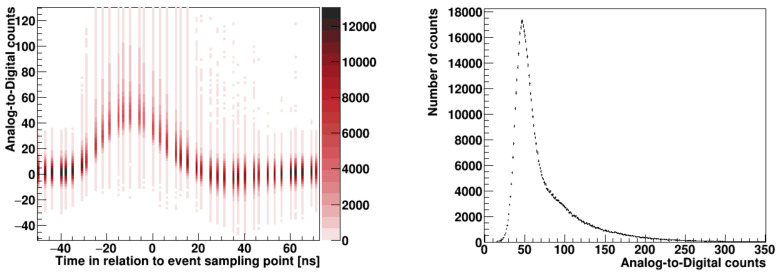
Pulse shape reconstructed with the Landau–Gauss distributions of the charge at each sampling point (**left**) and one selected distribution for the peak of the pulse (**right**).

The reconstructed pulse for the real proton–proton collisions is shown in the form of a two-dimensional histogram in Figure 7 (left). Each sample point contains a Gauss–Landau distribution of Analogue-to-Digital counts. To reconstruct the original shape, a Gauss–Landau distribution for each point has to be fitted. The pulse amplitude corresponds to the most probable value of the Gauss–Landau distribution that depends on the average particle energy and the sensor depletion depth.

When the new optimized configuration found in the previous section was tested in one of the VELO sensors, experimental studies have shown that this improvement not only leads to a shape of the signal with the lower R but also reduces the temperature of the sensor by 1 ${}^{\circ }$C and the power dissipation by around 3%. Moreover, a month before the end of Run 2, the improved configuration was incorporated into the detector set-up, and the efficiency test was carried out. The temperature of the sensor readout electronics was lower by over 1 ${}^{\circ }$C when the improved configuration was used. The conclusion of these tests was that the impact of the irradiation caused the sensor to slightly change the properties of the analogue readout, such that its nominal configuration in terms of the pulse shape condition was no longer optimal. The hardware readout optimization procedure presented in this paper can also be applied in the upcoming VELO upgrade I. Power dissipation is directly linked to the risk of the thermal-runaway effect, where the currents, after exceeding a certain threshold, exponentially grow and may result in the sensor breakdown. The reduction in the temperature by 1 ${}^{\circ }$C in the upgraded detector can be a very valuable step in increasing the resilience of the detector to radiation damage effects. It gives more operational freedom, for instance, the possibility to raise the operating bias voltage and current.

## 4. Conclusions

The optimization of the VELO readout system was performed. The advantages resulting from the modification of the electronic settings were two-fold. The reduction in the power consumption of the electronic chip to decrease the temperature is desirable as it gains the safety margin to the point of thermal runaway. The second advantage is related to reducing the spill-over hits sent for processing to the LHCb trigger system and enhancing hit and track reconstruction efficiency. Both of the advantages mentioned above were achieved in the studies presented in this paper. A similar procedure may benefit other silicon detectors operating at an extremely high radiation environment composed of mixed fast hadron fields. In particular, the upgraded Upstream Tracker can benefit from an optimization procedure of the readout ASIC SALT [38], which is similar to Beetle and features the analogue response allowing the pulse shape to be reconstructed. The instantaneous luminosity of the further Run 3 and 4 of LHCb is planned to be approximately five times higher than the luminosity seen in the LHCb detector so far. Thus, the presented studies and developed analysis techniques may play a crucial role in keeping the UT detector operational to the end of Run 4. The necessity of keeping the temperature of the readout system as low as possible will determine further research on this matter. Perhaps the idea behind the studies is even more valuable and reduces the power consumption of silicon detectors that can be by achieved operating in vacuum in other high energy physics experiments.

Finally, the pulse-shape reconstruction technique based on the machine learning algorithm can be applied easily for other silicon detectors featuring analogue readout chips by following the procedure described in the paper. It has several advantages with respect to the classical approach based on fitting complex models based on analytical functions. The complexity is hidden in the neural network itself and is of no concern for the user. This poses no problem, since most of the parameters fitted to any analytical functions used to model the pulse shape are nuisance parameters anyway. Moreover, the application of the trained neural network in the simulation code is much more straightforward. In particular, the precision problem of the fitted parameters, which may differ by orders of magnitude in analytical function models, is not present.

## Figures and Tables

**Table 1:** Overview of the different front-end configuration settings used. The numbers correspond to the curves presented in Figure 6.

	Configuration								
Parameter		Default	1	2	3	4	5	6	7
$I_{\mathrm{pre}}$	[μA]	600	800	640	496	400	600	536	600
$I_{\mathrm{sha}}$	[μA]	80	80	80	200	200	80	112	80
$V_{\mathrm{fp}}$	[mV]	510	600	600	600	700	510	510	510
$V_{\mathrm{fs}}$	[mV]	150	300	400	300	600	150	100	140
$I_{\mathrm{buff}}$	[μA]	80	80	80	80	80	80	80	80
$I_{\mathrm{currbuff}}$	[μA]	824	744	744	744	744	744	744	744

## Data Availability

Data sharing does not apply to this article.
